# A lymphatic malformation with a “Lympholith” and encasing neurovascular structures in the arm of a 2‐year‐old child: A case report

**DOI:** 10.1002/ccr3.6608

**Published:** 2022-11-19

**Authors:** Samit Sharma, Aadesh Rayamajhi, Jayan Man Shrestha

**Affiliations:** ^1^ Department of Plastic Surgery and Burns Tribhuvan University Teaching Hospital Kathmandu Nepal; ^2^ Institute of Medicine, Maharajgunj Medical Campus Tribhuvan University Kathmandu Nepal

**Keywords:** lymphatic malformation, lympholith, neurovascular structures

## Abstract

A 2‐year‐old child presented with a lymphatic malformation (LM) in the arm and the axilla. The neurovascular structures encased by the LM were preserved during the excision surgery, and a “lympholith” was found within a cyst of the LM.

## Graphical abstract

Lymphatic malformation (LM) in the arm can extend into deeper planes to encase the vital neurovascular structures. A “lympholith” can occur in the LM and could suggest a low‐flow/ hypodynamic LM.

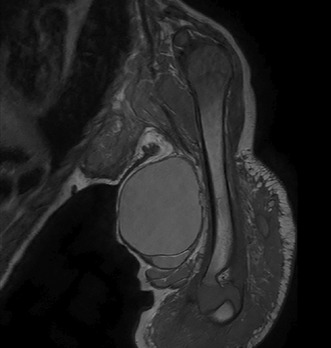

## 1 INTRODUCTION

Lymphatic malformations (LMs) or lymphangiomas are rare benign cystic masses. They are caused by abnormal development of the lymphatic system. Cyst formation occurs due to lymphatic accumulation from abnormal or absent communications between the central venous sacs and the peripheral lymphatic system.^1^, ^2^ LMs are commonly located in the head and neck region (75%) and the axilla (20%). They are rarely found in the mediastinum, retroperitoneum, inguinal region, breast, and pelvic cavity.^3^ The incidence of LM is estimated to be 1:6000 to 1:16000 live births.^4^ Approximately 50% are evident at birth, and 80% are detected by 2 years of age.^5^ Some are diagnosed prenatally as well.

Lymphatic malformations tend to be soft, spongy, nontender masses. The clinical features vary based on the location and size and can range from local swelling resulting in a superficial mass to a large area of lymphatic channel infiltration resulting in elephantiasis.^6^ LMs usually grow slowly and steadily, but the growth can be problematic causing functional impairment of nearby structures or organs and disfigurement of affected areas.

Herein, we report a case of a 2‐year‐old child with lymphatic malformation encasing the neurovascular structures in the arm and with a stone‐like content within a cyst, which we would prefer to refer to as a “lympholith” similar to a phlebolith found in veins in other conditions.

## 2 CASE PRESENTATION

A 2‐year‐old female child from southern Nepal was brought to the outpatient plastic surgery clinic with the complain of a large swelling in the left arm and axilla since birth. The swelling was progressively increasing in size with no associated pain or skin changes. The child did not have fever or loss of weight. There was no history of trauma or swelling in other parts of the body.

Examination revealed a large swelling extending from the axilla to the proximal forearm. The swelling was soft, spongy, nontender, and without prominence of superficial veins. Pulsations could not be palpated over the swelling, and no bruits were heard on auscultation.

**FIGURE 1:**
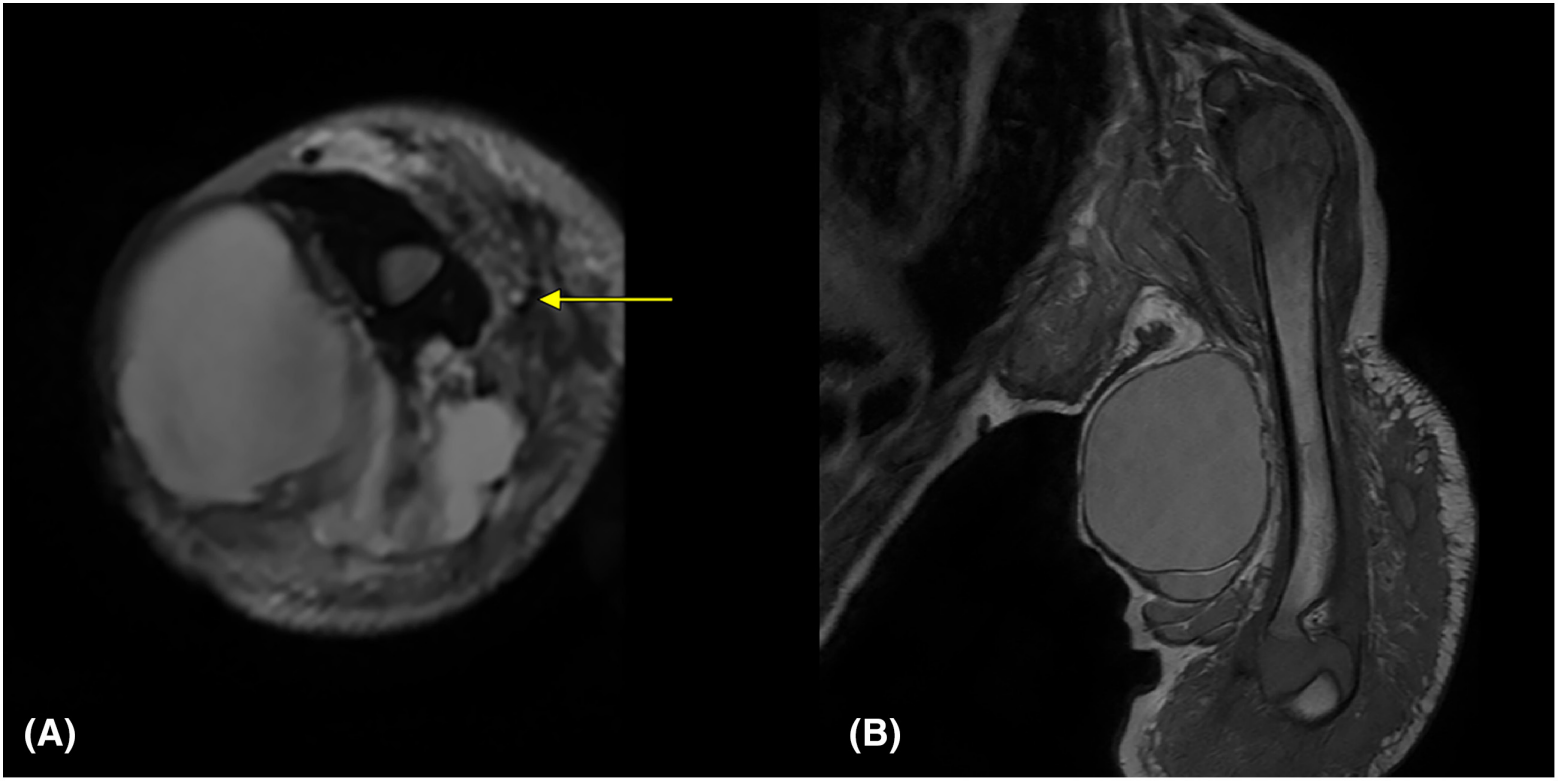
(A) T2 high signal intensity multicystic lesions in intramuscular compartment of proximal arm on axial section; neurovascular bundles (arrow) are displaced anterolaterally at this level. (B) Heterogenous mixed T1 low and high signal intensity lesions in intramuscular compartment of proximal arm on coronal section; adjacent cortex of humerus appears normal.

Magnetic Resonance Imaging (MRI) showed a complex multiloculated mass in the medial aspect of arm encasing the neurovascular bundle, extending to the axilla proximally and elbow and forearm distally. Cystic components of different sizes were noted within the mass with variable thickness septations. Some components showed T1 and T2 hyperintense content with low signal intensity in fat suppressed T2‐weighted images, while the others showed variable signal intensities. There was associated edema in the subcutaneous plane in the arm and the axilla. Humerus showed normal cortical and marrow signal intensities without any evidence of bony erosion or destruction (Figure 1).

**FIGURE 2:**
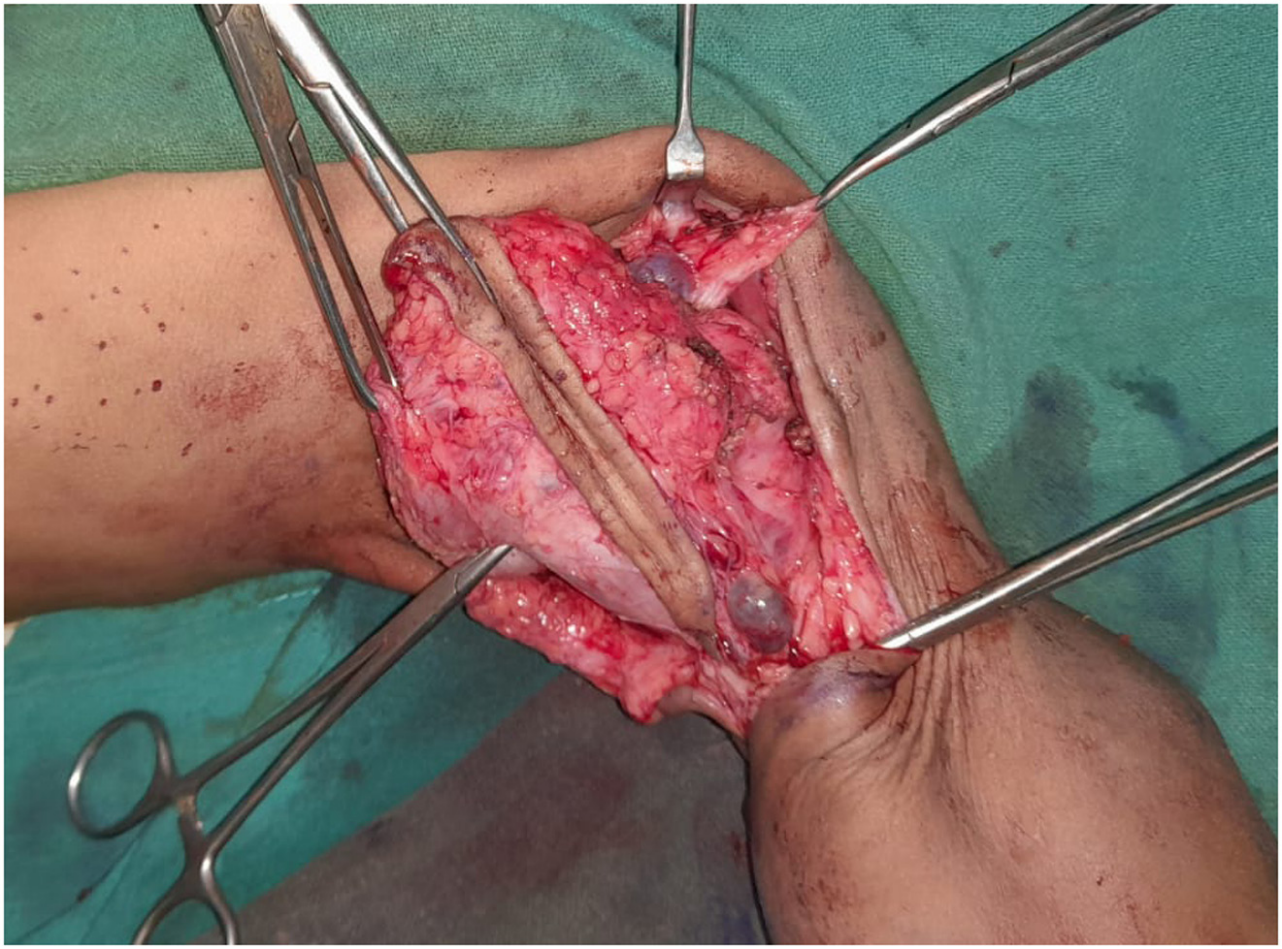
Dilated lymphatic channels seen intraoperatively.

**FIGURE 3:**
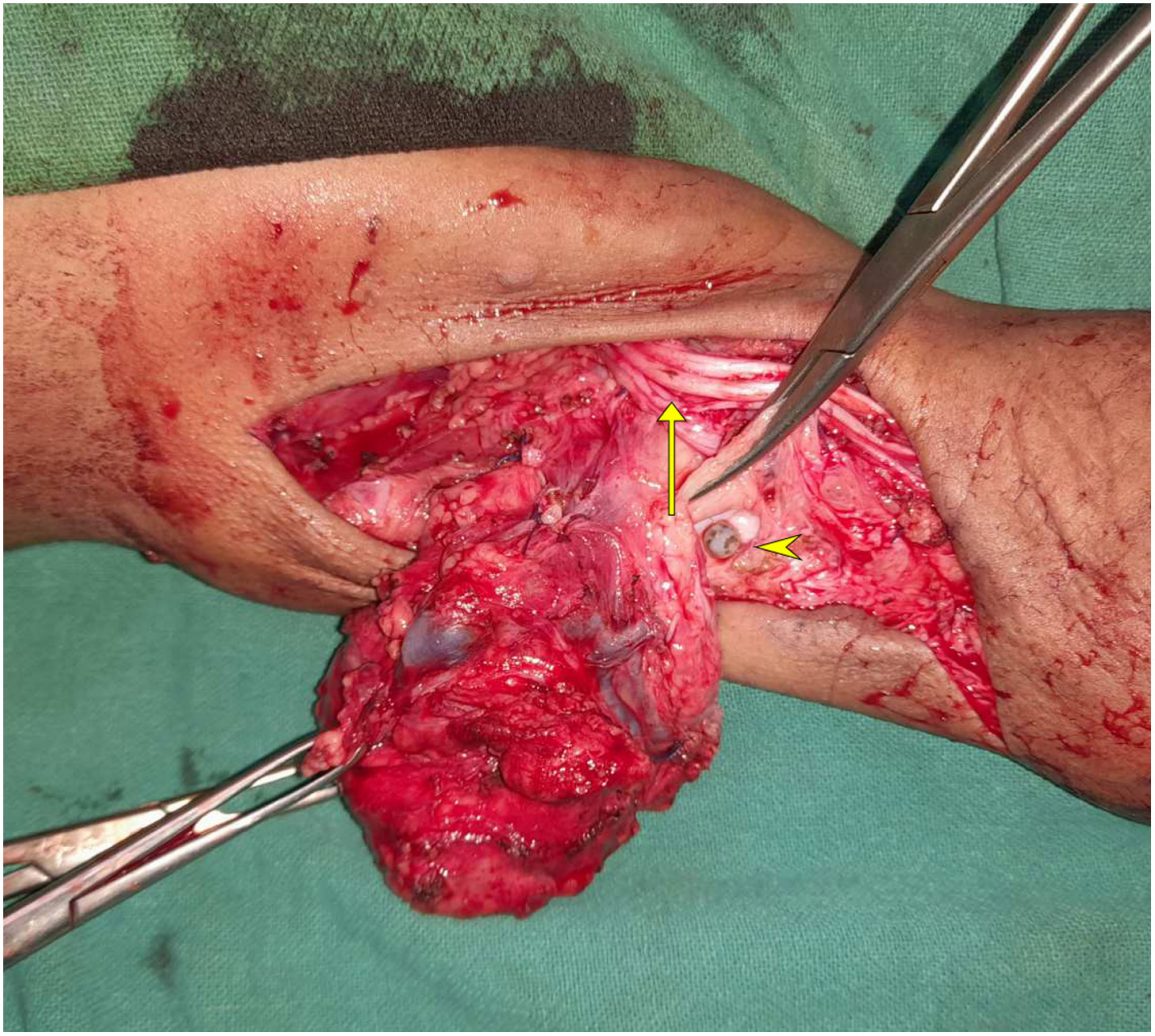
Intraoperative finding of a “lympholith” (arrowhead) and neurovascular structures being displaced anterolaterally (arrow).

A debulking surgery was planned under general anesthesia. The swelling was approached through a medial arm incision. Dilated lymphatic channels could be seen intraoperatively (Figure 2). There were two most notable findings:

1. Finding of a stone‐like structure within a cyst – “lympholith” (Figure 3)
2. The mass was encasing the median nerve, the ulnar nerve, and the brachial artery and had pushed these structures anterosuperiorly (Figure 3).

**FIGURE 4:**
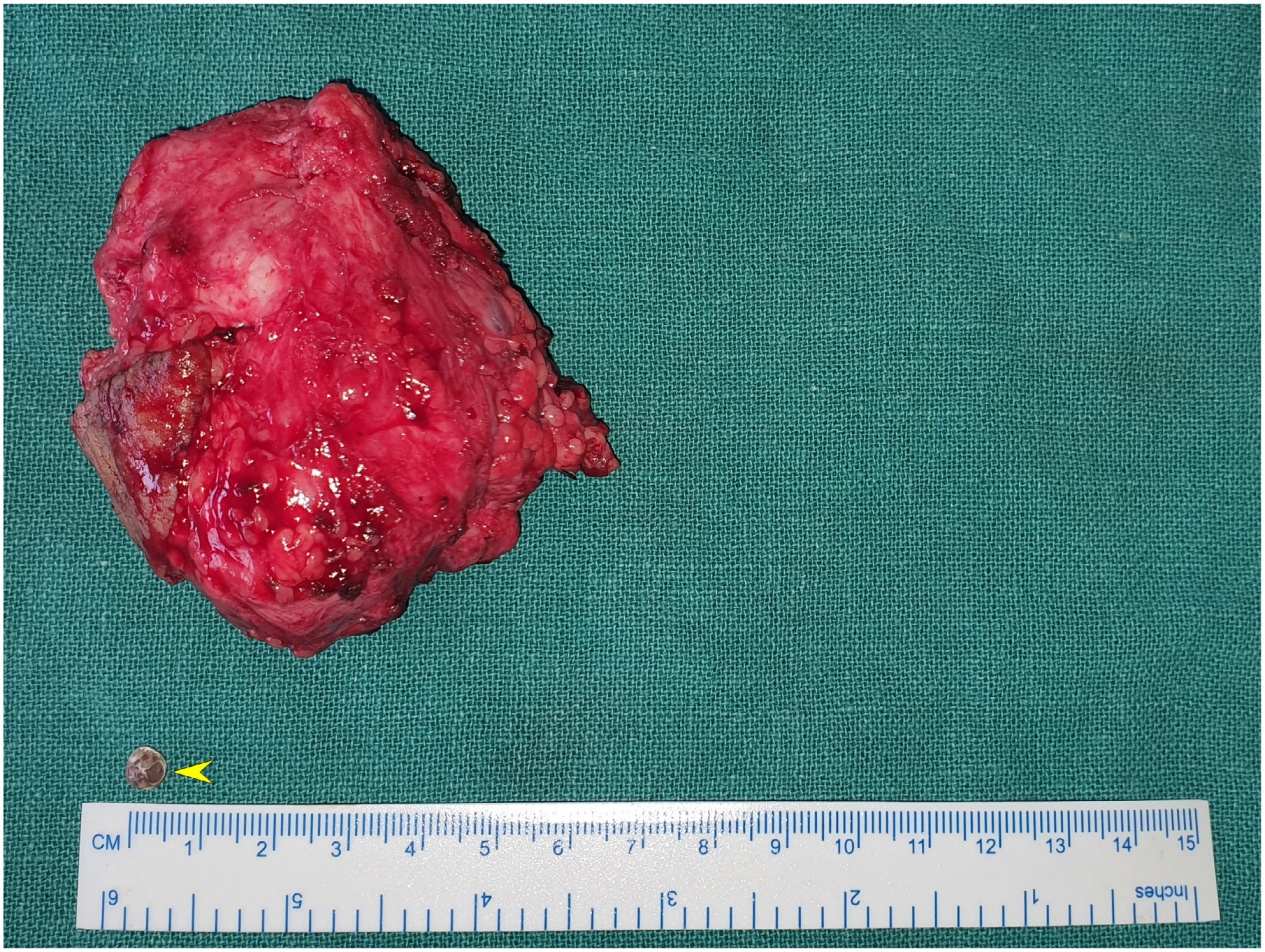
Excised surgical specimen measuring 6.0 × 7.0 cm and a lympholith measuring 0.5 × 0.5 cm (arrowhead).

**FIGURE 5:**
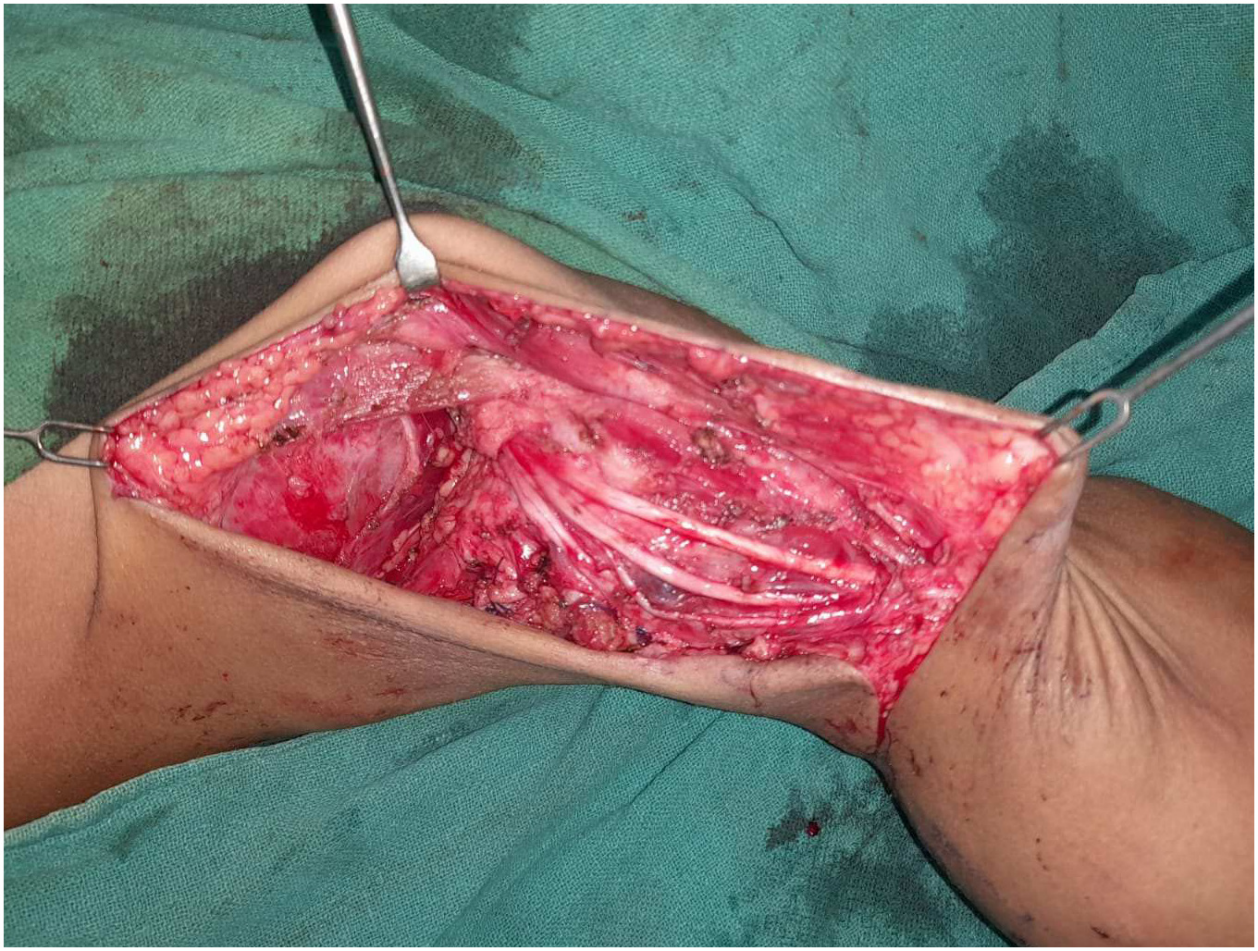
Intact neurovascular structures seen at the end of the surgery.

**FIGURE 6:**
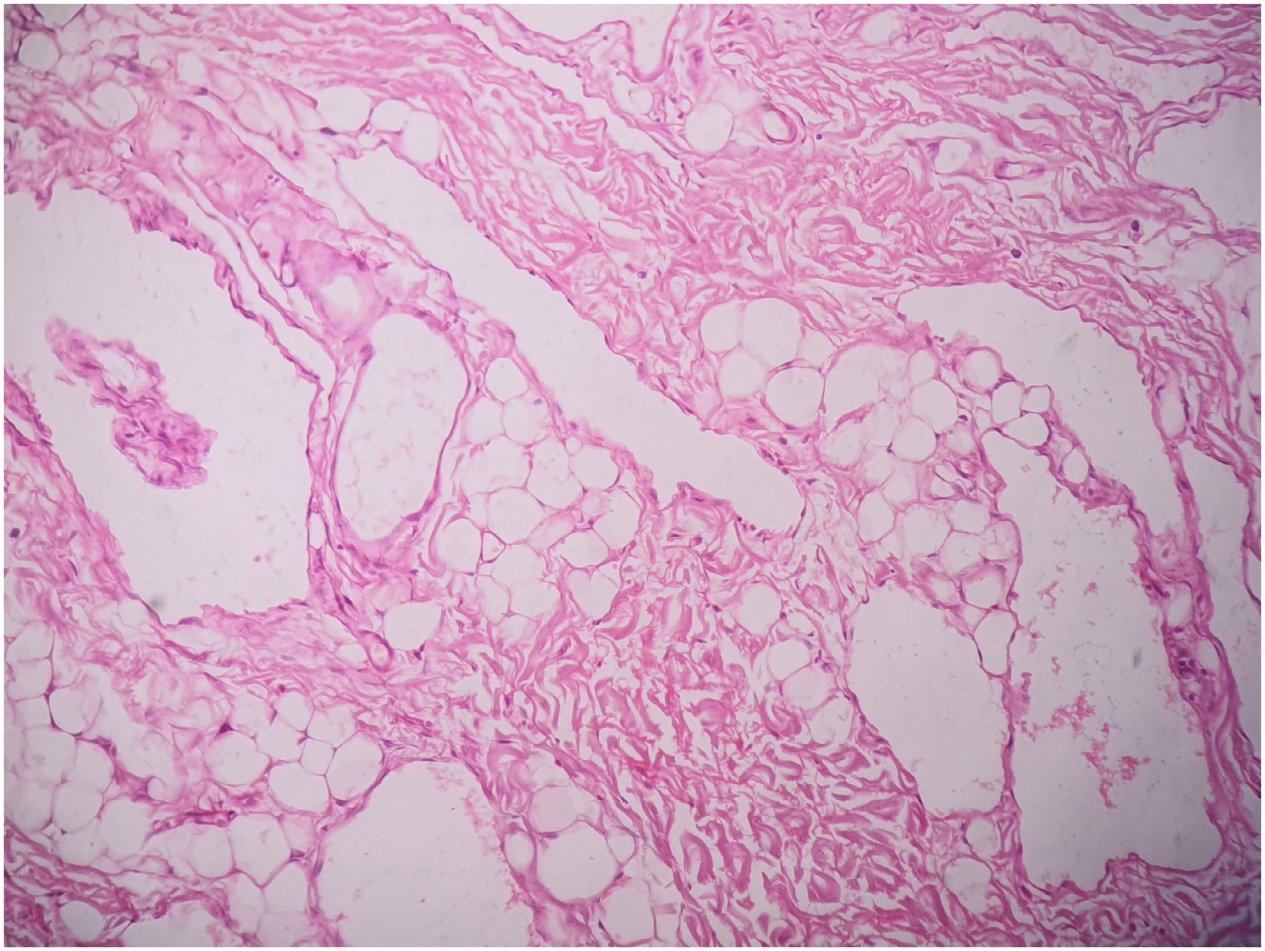
Histopathology of the excised specimen.

The excised surgical specimen measured 6.0 × 7.0 cm and the lympholith measured 0.5 × 0.5 cm (Figure 4). With meticulous and careful dissection of the mass, the neurovascular structures were safeguarded and preserved (Figure 5). Due to tedious dissection around the neurovascular structures and prolonged surgery for a 2‐year‐old child, distal portion of the lesion was planned for excision at a later setting. Histopathology of the excised specimen revealed multiple dilated lymphatic channels lined by bland looking endothelial cells (Figure 6) consistent with a lymphatic malformation. On D2‐40 immunostaining, the lymphatic endothelial cells were positive.

## 3 DISCUSSION

According to International Society for the Study of Vascular Anomalies (ISSVA), vascular anomalies can be classified into tumors (endothelial cell proliferation – benign, borderline, or malignant) or malformations (structural or morphologic anomalies resulting from faulty embryonic morphogenesis). Vascular malformation (VM) can further be simple or combined. Under simple VM lie capillary, lymphatic, venous, and arteriovenous malformations. Common (cystic) lymphatic malformation is classified under the heading of LM, which is further classified as macrocystic, microcystic, or mixed.^7^ Various synonyms and forms of LM are found to be used in the literature such as lymphangioma, cystic hygroma, cavernous lymphangioma, and cystic lymphangioma, but we have used the terms suggested by the ISSVA for uniformity and to avoid confusion. Macrocystic cysts are cysts greater than 2 cm with clear boundaries and the microcystic type is the ones with diameter less than 2 cm and with ill‐defined boundaries. The macrocystic type (previously known as cystic hygroma) develops more frequently in the neck, and the microcystic type (previously known as lymphangioma) develops more frequently in the oral cavity and cheek.^8^, ^9^, ^10^

Vascular malformations can also be classified as low‐flow and high‐flow according to their hemodynamic characteristics.^11^, ^12^ The low‐flow VMs are composed of non‐arterial components and include capillary, venous, and lymphatic malformations, whereas the high‐flow VMs are composed of arterial components, including arteriovenous malformations and arteriovenous fistulas.^11^, ^13^, ^14^

Lymphatic malformations account for 5%–6% of all benign soft tissue masses in children.^2^, ^8^, ^15^ They are rare, benign, low‐flow vascular anomaly arising from malformation consisting of dilated lymphatic vessels in soft tissues. Aberrant lymphatic morphogenesis results in failure of communication with the venous system, rendering lymphatic malformation isolated from the normal lymphatic system.^14^, ^16^, ^17^ The cysts in LMs are usually multilocular because they occur after the arrest of lymphatic system development at early stages of embryogenesis,^18^, ^19^, ^20^, ^21^ as was seen in our case.

Lymphatic malformations usually present as asymptomatic masses with parental aesthetic concern. Even large lesions may not cause symptoms as they grow within an overlying elastic skin and subcutaneous tissue.^15^ The lesions transilluminate and are not necessarily compressible.^16^ They can be predisposed to sudden growth as a response to immunologic stimuli (most frequently, a common cold), hemorrhage, or infection. Hemorrhage can lead the lesions to be painful with a bluish or purplish hue simulating a venous malformation and can also be painful, indurated, or erythematous due to infection.^16^ They can sometimes present with compression effects such as respiratory distress when located in the neck. LMs usually do not undergo spontaneous regression. Though regression has been reported in 1.6%–16% of cases, recurrence is most likely to occur.^10^

Though ultrasonography may be the first‐line imaging modality for the diagnosis of LMs, contrast‐enhanced MRI is the preferred imaging. MRI is of value for deep and extensive lesions, providing valuable information relating to size, extent, tissue involvement, and pre‐procedural planning.^16^ Pre‐operative imaging with MRI was very useful in our case as the LM was encasing the adjacent neurovascular structures.

The main treatment options for LMs are sclerotherapy, surgery, laser therapy, radiofrequency ablation, or a combination of these modalities. Minimally invasive modalities are of use in situations when there are contraindications for anesthesia, in high‐risk surgery or to down‐stage the size of the lesion.^22^ Most moderately sized macrocystic LMs can be treated with sclerotherapy. However, sclerotherapy requires multiple sessions to be effective when the lesions are extensive and the agents can have their own side effects.^23^

Surgical excision remains the mainstay of treatment for giant macrocystic lesions. The aim of surgery is to preserve/regain the function of an affected area and to prevent disfiguring complications. LMs most commonly involve the subcutaneous tissue, but can extend deeper insinuating deeper structures as in our case. Though most of the LMs occur in the head and neck region and the axilla, they can also occur in other parts of the body such as the arm and encase vital structures. In our case, the lesion involved most of the arm extending up to the axilla proximally and elbow region and proximal forearm distally. Surgeons should be mindful to preserve the vital neurovascular structures during excision. From our case it can be seen that LMs in the limbs can disregard anatomic and fascial boundaries and can involve multiple tissue planes not just limiting themselves to the subcutaneous plane. The damage to the median and ulnar nerves and the brachial artery could be detrimental to the patient, and hence, meticulous dissection was carried out to preserve those structures and their function in addition to preventing the mass effect, the swelling might have in the future. This makes such surgeries tedious and time‐consuming yet challenging and worthwhile. The proximity and adherence of the LM to important and vital structures may at times warrant only partial removal or debulking of the lesion. Moreover, very difficult cases may be staged for a later setting after partial removal/debulking as was done in our case.

Another important finding in our case was that of a stone‐like content in one of the cysts, which we want to refer to as a “lympholith.” Though some cases of phleboliths and angioliths have been reported, we could not find any literature on “lympholith” when searched on popular databases. Angiolithiasis is vascular‐based formation of calculi and an angiolith, a calcified thrombus within venules, veins, or sinusoidal vessels of a hemangioma. Phleboliths are intravascular stone formation in the veins. Pathogenesis of angioliths and phleboliths is probably the same; formation of intravascular thrombosis under the influence of proliferating fibroblasts, which organizes and becomes secondarily mineralized.^24^ Moreover, the contact of lymphatic stream with necrotic cells or infection of the tissues in the neighborhood of the lymphatic vessels can trigger lymphatic thrombosis.^25^ “Lympholiths” perhaps exhibit similar pathogenesis; intra‐lymph vessel/channel thrombosis with secondary mineralization. Further studies are required to corroborate the exact pathogenesis. Many studies have correlated phlebolith as a marker of slow‐flow venous malformation.^26^, ^27^ Similarly, lympholith could also be a marker of a slow‐flow/hypodynamic LM. Further studies are needed to investigate this.

## 4 CONCLUSION

Lymphatic malformation can rarely occur in the arm and involve multiple tissue planes extending deeper to encase neurovascular structures, which the surgeon should be mindful of during the dissection for surgical excision/debulking. Lympholiths can present in an LM and could suggest a slow‐flow or hypodynamic LM.

## AUTHOR CONTRIBUTIONS

SS and AR: involved in concept, collecting information, manuscript writing, and participated in literature review and edited the draft. SS and JMS: involved in patient care team and also independently reviewed the manuscript. SS, AR, and JMS: re‐edited the draft and reshaped it into this manuscript. All authors approved the final version of the manuscript and agree to be accountable for all aspects of the work in ensuring that questions related to the accuracy or integrity of any part of the work are appropriately investigated and resolved.

## CONFLICT OF INTEREST

The authors declare that there is no conflict of interest regarding the publication of this paper.

## ETHICAL APPROVAL AND CONSENT TO PARTICIPATE

Need for ethical approval waived. Consent from the patient's father deemed to be enough.

## CONSENT

Written informed consent was obtained from the patient's father for publication of this case report and any accompanying images. A copy of the written consent will be available for review if asked by the editor‐in‐chief of this journal.

## Data Availability

Not applicable.
